# Gaze-driven placement of items for proactive visual exploration

**DOI:** 10.1007/s12650-021-00808-5

**Published:** 2021-11-11

**Authors:** Shigeo Takahashi, Akane Uchita, Kazuho Watanabe, Masatoshi Arikawa

**Affiliations:** 1 Department of Computer Science and Engineering, University of Aizu, Aizu-Wakamatsu, 965-8580 Japan; 2 Department of Computer Science and Engineering, Toyohashi University of Technology, Toyohashi, 441-8580 Japan; 3 Graduate School of Engineering Science, Akita University, Akita, 010-8502 Japan

**Keywords:** Gaze-driven interaction, Search context, Optimization, User evaluation

## Abstract

Recent advances in digital signage technology have improved the ability to visually select specific items within a group. Although this is due to the ability to dynamically update the display of items, the corresponding layout schemes remain a subject of research. This paper explores the sophisticated layout of items by respecting the underlying context of searching for favorite items. Our study begins by formulating the static placement of items as an optimization problem that incorporates aesthetic layout criteria as constraints. This is further extended to accommodate the dynamic placement of items for more proactive visual exploration based on the ongoing search context. Our animated layout is driven by analyzing the distribution of eye gaze through an eye-tracking device, by which we infer how the most attractive items lead to the finally wanted ones. We create a planar layout of items as a context map to establish association rules to dynamically replace existing items with new ones. For this purpose, we extract the set of important topics from a set of annotated texts associated with the items using matrix factorization. We also conduct user studies to evaluate the validity of the design criteria incorporated into both static and dynamic placement of items. After discussing the pros and cons of the proposed approach and possible themes for future research, we conclude this paper.

## Introduction

**Fig. 1:**
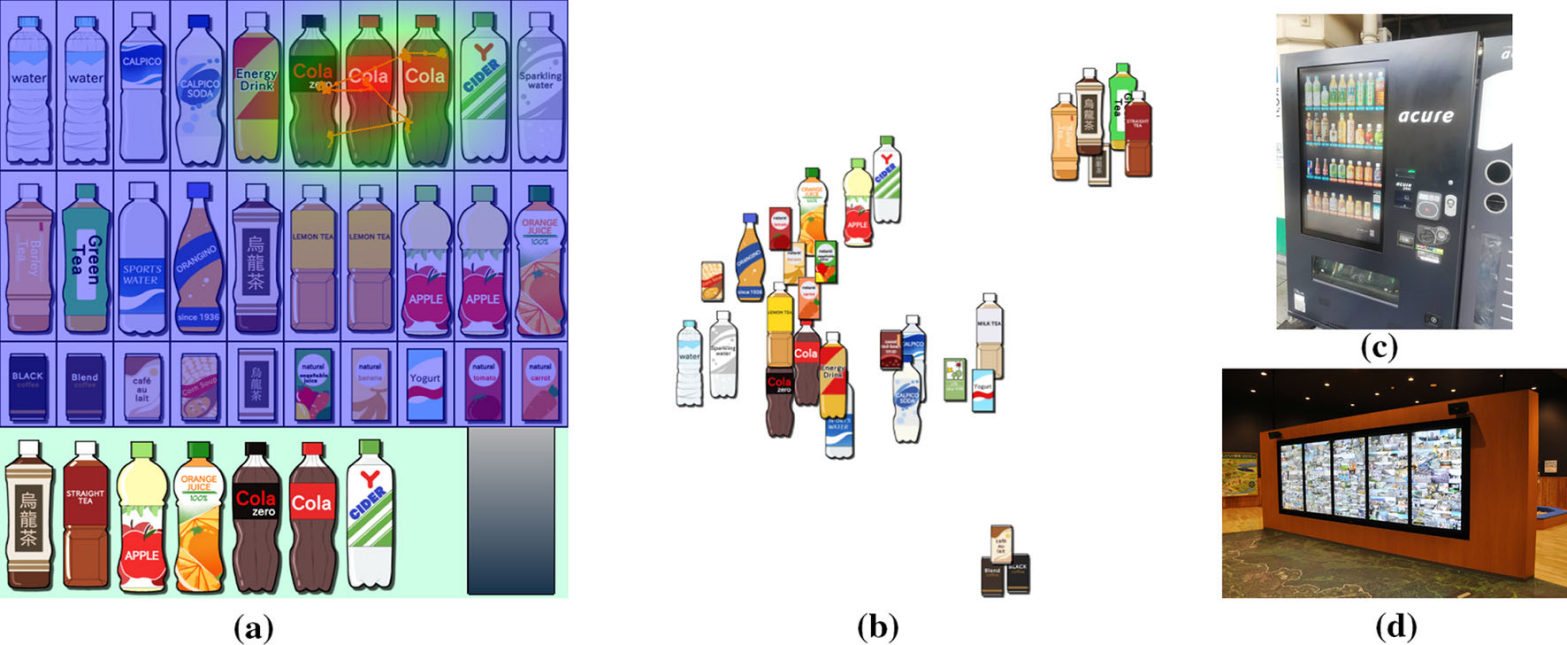
**a** Gaze-driven placement of drinks in a virtual vending machine. The heatmap and gaze plot represent how visual attention is directed. **b** Context map for describing association rules among drinks.** c** Contemporary digital signage displays on a vending machine.** d** Information wall in a learning facility (courtesy of Sustaina Kyoto, Kyoto, Japan)

Item layout significantly influences the ability to attract the visual attention of viewers when advertising and encouraging the extensive use of products. Digital signage technologies successfully help us enhance such visual representation in this era of multimedia communication. Typical examples include digital displays of products on contemporary vending machines (Fig. 1c) and the exhibition of visual art on information walls (Fig. 1d). While this style of visual presentation successfully facilitates improved placement of items for enhanced impact, the strategy for arranging the associated digital contents still needs to be further investigated.

Optimal placements of items have been explored for a long time, even in our daily life. For example, the layout of items significantly impacts sales results in retail stores, such as grocery shops and supermarkets. In this case, we need to attract customers with the layout of products by considering general co-purchasing rules. This is because we usually do not have any prior knowledge about the specific preferences of the respective customers nor do we instantly change the placements of products themselves even when we know their favorites. The layout strategy is different in online shops, in which the layouts of products change according to the preferences of the customers. This is accomplished by accumulating co-purchasing data as a product-customer matrix and extracting characteristic co-purchasing patterns to meet the requirements of each customer.

Employing digital display devices poses another unique technical problem. This is because we can freely change the placement of items without sufficient knowledge about the preferred choices of each customer. Thus, we need to provide users with an opportunity to explore their favorite items by suggesting related items even when they do not explicitly identify which specific items are the best. This visual exploration should be sufficiently proactive in that the item placement faithfully simulates the ongoing search context of an individual viewer. In this case, the number of items prepared for exhibition usually exceeds the number of cells in the digital display. This means that we have to carefully select a set of visible items by replacing unwanted items with possibly preferred ones according to the search context. This consideration motivated us to pursue a novel approach for dynamically rearranging item layouts by interacting with the customers through the display.

In this study, we employ an eye-tracking interface to explore the personal preferences of viewers by respecting the underlying context in the search for items of interest. The eye-tracking technology helps us infer the preferences of the viewers by analyzing how they focus their visual attention on the layout of items. In practice, eye-tracking input devices have become popular for controlling multimedia contents, including video games, interactive controllers, and communication tools. This type of visual interaction is an important trigger to dynamically update the digital display of items by respecting the underlying search context of the viewer. Besides, we also try to replace current sets of items with favorite ones specific to the viewer by predicting the search path in the configuration space of the items.

In this paper, we advance our previous work (Takahashi et al. 2020) by conducting extensive user studies to adequately justify our design criteria in the placement of items. In addition to this novel contribution, we implement sophisticated association rules among items to dynamically update their placement by incorporating text mining techniques based on matrix factorization.

In this paper, we first introduce the static placement of items as an optimization problem and important aesthetic criteria as constraints. We then describe the dynamic version so that we can rearrange the items according to the search context of viewers. To guide dynamic item arrangement, we employ an eye-tracker that takes spatiotemporal eye gaze distribution as input. We also construct a context map to retain the association rules among items by applying topic-based mining techniques to annotated texts associated with the items. This allows us to instantly infer the following favorite items from the most focused one by investigating its spatial neighbors on the context map. We demonstrate the feasibility of the proposed approach by simulating a virtual vending system and a digital information wall. We conduct extensive user studies to evaluate the validity of the respective layout constraints incorporated in our approach. This includes an investigation of design criteria for both static and dynamic placement through online questionnaires and eye-tracking experiments. We also compare association rules among items obtained using our context map and those reproduced by conventional recommendation systems based on co-purchasing data.

In summary, our technical contribution lies in developing a new scheme for proactively exploring favorite items among possible choices through digital signage technology. More specifically, our advancements can be listed as follows:

- Formulating the optimal static placement of items quantitatively as a constrained optimization problem
- Developing gaze-driven interaction to improve the dynamic placement of items by respecting the context in the search for favorites
- Introducing matrix factorization into topic-based text mining to enhance the quality of association rules among items
- Conducting user studies to justify the proposed design criteria for the placement of items

The remainder of this paper is organized as follows. We first provide a survey of previous work related to our approach in Sect. 2. We then formulate the static placement of items as a constrained optimization problem in Sect. 3. We detail how we can extend our formulation for static placement to the dynamic version by taking as input spatiotemporal eye gaze distribution. We also introduce text mining techniques to construct a context map so that we can infer a set of association rules among items by respecting the underlying search context. After demonstrating our experiment results, we justify the design criteria through user studies and discuss the possible limitations and future extensions of our approach in Sect. 6. Finally, we conclude this paper in Sect. 7.

## Related work

We survey previous studies related to ours by categorizing them into pattern layout optimization, gaze-driven interaction, and machine learning techniques.

### Pattern layout optimization

Designing aesthetic layouts of visual elements poses a vital problem in enhancing the readability of visual information. This is true for visual exhibitions based on digital signage technologies, including interactive guide maps (Li et al. 2020) and large information walls (Chen et al. 2021). Color map design has been another essential target for optimization (Wang et al. 2021) to improve perceptual experiences in visual interaction.

On the other hand, to design schematic pattern layouts, mathematical programming techniques are advantageous; they effectively facilitate the clear distinction between soft and hard constraints. For example, we can employ *linear programming* (LP) to draw bundled parallel coordinate plots (Zhou et al. 2008), consistently distorted 2D residential maps (Maruyama et al. 2019), and 3D urban maps free of route occlusions (Hirono et al. 2013). We can also apply integer programming (IP) to the design of orthogonal networks (Eiglsperger et al. 2000; Yoghourdjian et al. 2016), schematic metro maps (Nöllenburg and Wolff 2011; Wu et al. 2013), and Sankey diagrams (Zarate et al. 2018). The combination of linear and quadratic programming has been successfully introduced to eliminate unwanted conflicts among visual elements (Meulemans 2019).

Constrained optimization techniques were also employed to align visual elements in a grid layout. Gomez-Nieto et al. (2013) formulated the problem of arranging thumbnail video snapshots on a grid as an IP problem and later combined it with multidimensional projection techniques (Gomez-Nieto et al. 2016). Strong and Gong (2014) invented a Self-Sorting Map for aligning multimedia elements by referring to their mutual dissimilarity. Liu et al. (2018) developed a constrained *multidimensional scaling* (MDS) solver to faithfully retain the underlying grid in the placement of items. Pan et al. (2019) introduced an approach for generating a tree-based layout of items by adaptively partitioning the screen space.

### Gaze-driven interaction

Eye-tracker equipment has been commonly employed to evaluate the quality of visual information and its associated visual interfaces (Andrienko et al. 2012). This research trend inspired the development of gaze-based interactive systems for the analysis of the temporal transition of *areas-of-interest* (AOIs) as trees (Tsang et al. 2010), river styles (Burch et al. 2013), space-time 3D cubes (Kurzhals et al. 2014), circular transition diagrams (Blascheck et al. 2013), and video editing operations (Jain et al. 2015). Burch et al. (2020) recently presented an exciting concept of an attention map, in which a set of visually attended regions are cropped and rearranged in a tag-cloud style. Readers can refer to a detailed survey on visualization of eye-tracking data (Blascheck et al. 2014).

Recently, eye-trackers have become the standard interface for interacting with visual displays due to their high cost-effectiveness. Video games enabled by eye-tracking technology (Gomez and Gellersen 2019; Smith and Graham 2006; Sundstedt 2010) are a typical example. Gaze-driven interaction has also been introduced to enhance the understanding of data for possible use in information visualization (Streit et al. 2009), graph visualization (Okoe et al. 2014), visual analytics (Silva et al. 2019), and annotated map visualization (Göbel et al. 2018). Eye-tracking technology is also beneficial for exploring virtual 3D space, including urban exploration (Baldauf et al. 2010) and fly-through travels (Qian and Teather 2018). Duchowski (2018) provides a survey of state-of-the-art techniques for gaze-based interactions.

Although several user studies have explored the potential design of the gaze-adaptive interface for visualization (Raptis et al. 2017; Steichen et al. 2014a, b), practical system design for the support of digital signage technology remains to be pursued. Our challenge may have some connection with a gaze-driven interface for optimizing the layout of floor plans (Alghofaili et al. 2019), as this technique employs a relatively simple energy-minimization approach. Furthermore, respecting the underlying context of searching for superior objects is crucial, especially when presenting animated visual displays (Li et al. 2019). To the best of our knowledge, this work is the first to design a gaze-adaptive interface that respects the ongoing context in the search for items of interest.

### Machine learning techniques

Machine learning techniques often give us valuable insights into the relationships among target items by analyzing available data. Among them, *Topic Modeling* enables us to effectively discover important topics shared by a corpus of documents and often plays a crucial role in visualizing texts in terms of such extracted topics. Lee et al. (2012) introduced the topic analysis called *latent Dirichlet allocation* (LDA) (Blei et al. 2003) to develop an interactive system for visually understanding clusters of documents. In place of LDA, Kim et al. (2015) applied *non-negative matrix factorization* (NMF) (Kim and Park 2011; Lee and Seung 1999) to larger sets of documents in the context of interactive topic analysis and further enhanced the scalability of the system through the hierarchical representation of topics (Kim et al. 2020). A scheme called TopicLens (Kim et al. 2017) offered a lens interface for dynamically exploring document data, where *t-distributed stochastic neighbor embedding* (t-SNE) (van der Maaten and Hinton 2008) was employed as a 2D embedding algorithm together with NMF. El-Assady et al. (2018) addressed the problem of analyzing the thematic composition of document corpora and then enhanced it to implement an incremental hierarchical algorithm for the topic modeling process (El-Assady et al. 2019).

Matrix factorization techniques also serve as a basis for implementing recommendation systems (Koren et al. 2009) if we have easy access to extensive training datasets, such as customer-product matrices. In this study, we expect that our approach will effectively support the collection of such data while maximally respecting the search context of the users at the same time. This research compares topic analysis techniques based on LDA and NMF and employs the NMF-based approach as our text mining technique (Sect. 5).

## Optimizing static placement

In this study, we assume that items are arranged in a grid on a digital display. We formulate this layout as an IP problem in which a binary variable represents the existence of each item in a specific cell on a grid.

**Fig. 2:**
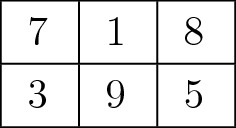
A $2 \times 3$ grid placement of numbers ranging from 1 to 9

### Basic formulation

Suppose that we place items on a $P \times Q$ grid of cells where $i (= 1,\ldots ,P)$ and $j (= 1,\ldots ,Q)$ correspond to the row and column IDs in a grid, respectively. Here, we introduce a binary variable, $X^{i,j}_k$, to represent the existence of the *k*-th item ($k = 1,\ldots ,N$) in the cell of the *i*-th row and *j*-th column on a grid. This implies that $X^{i,j}_k = 1$ if the (*i*, *j*)-cell contains the *k*-th item; otherwise, $X^{i,j}_k = 0$. In this setup, we need to prepare $P \times Q \times N$ binary variables, $X^{i,j}_k (i = 1, \ldots , P; j = 1, \ldots , Q; k = 1, \ldots , N)$, to completely describe the placement of items. Figure 2 shows a $P = 2 \times Q = 3$ grid of cells as our running example, where we place numbers ranging from 1 to $N = 9$. For example, we can mathematically express the placement of numbers in Fig. 2 as $X^{1,1}_7 = 1, X^{1,2}_1 = 1, X^{1,3}_8 = 1, X^{2,1}_3 = 1, X^{2,2}_9 = 1, X^{2,3}_5 = 1$, and the other binary variables, $X^{i,j}_k (i = 1,2; j=1,2,3; k=1,\ldots ,9)$, equal 0.

Here, we can impose several known constraints as equations and inequalities. First of all, a trivial constraint can be given by

$$\begin{aligned} \sum _{k=1}^N X^{i,j}_k = 1 \quad (i = 1,\ldots ,P; j=1,\ldots ,Q), \end{aligned}$$

for each cell because every grid cell retains only one single item out of *N* choices. We can introduce additional constraints as

$$\begin{aligned} L_k \le \sum _{i=1}^P \sum _{j=1}^Q X^{i,j}_k \le U_k \nonumber \end{aligned}$$

if we bind the number of appearances for the *k*-th item within $[L_k, U_k]$. For example, we can set $\sum _{i=1}^P \sum _{j=1}^Q X^{i,j}_k = 1$ if we place the *k*-th item only once on the $P \times Q$ grid.

### Design criteria for static placement

In addition to the basic formulation described above, we incorporate several criteria for designing the static placement of items as follows:

(S1): Aligning items of the same size in a specific row
(S2): Placing two items of the same category next to each other
(S3): Arranging the same items in a sub-matrix

These criteria were inspired by understanding the underlying rules in placing items in actual vending machines and information walls through observation. We will explain how we can implement each criterion in the remainder of this section. We will report the validity of these design criteria obtained from user studies later in this paper. Recall that, as described earlier, we have more items to exhibit than the number of available cells in the display. We determine which items should be visible in the optimization process (See Sect. 4.2).

*(S1) Aligning items of the same size in a specific row*

**Fig. 3:**
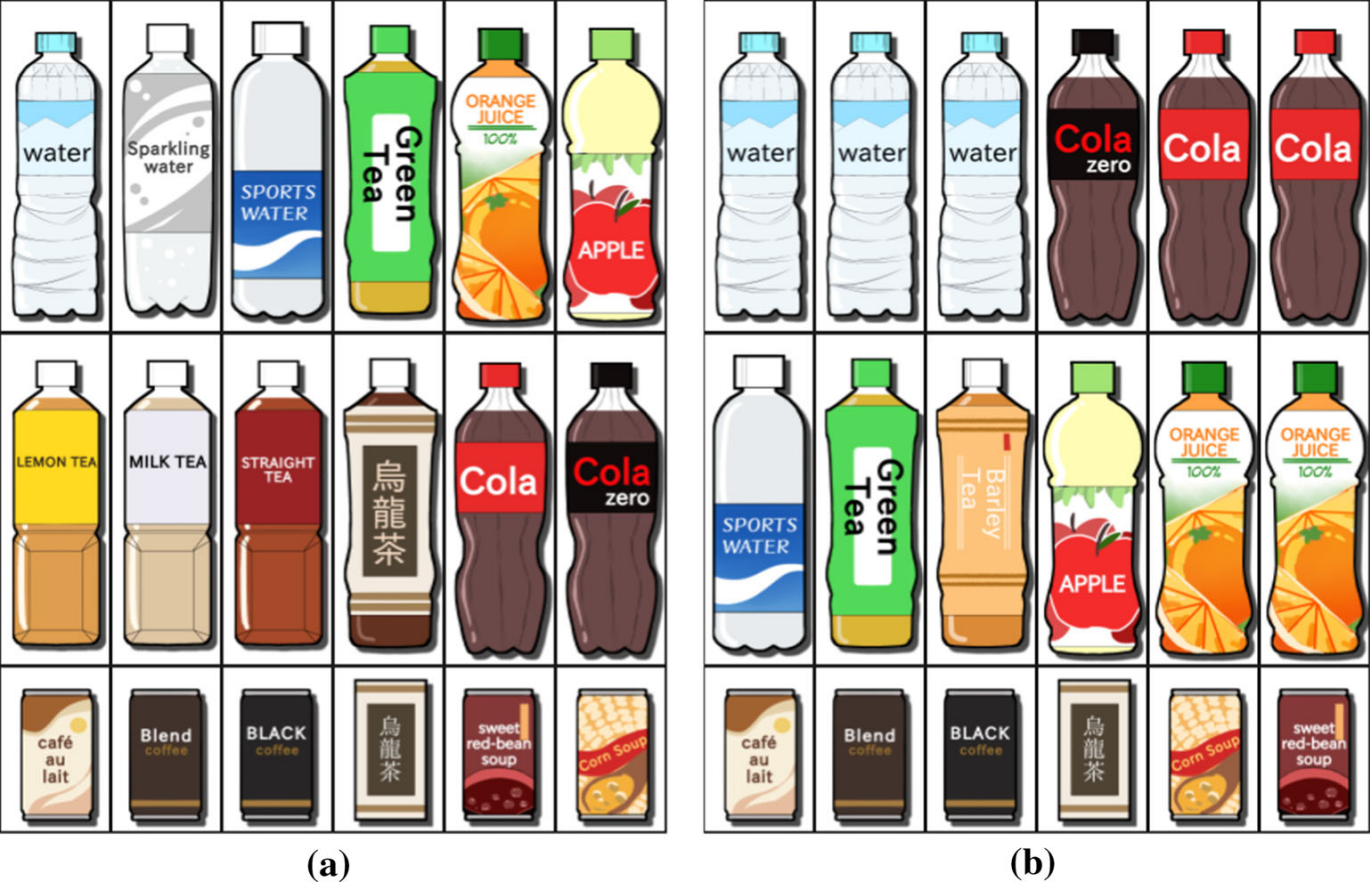
Optimizing the static placement of items.** a** Drinks of the same category are arranged next to each other.** b** Identical drinks are lined up in a row

The criterion (S1) helps us maximize space efficiency in the layout of items by arranging them compactly according to their sizes and has commonly been employed in the display interface in contemporary vending machines. This design rule can be accomplished by intentionally restricting the available cells for a specific item. For example, in the case of Fig. 2, we can fix 7 at (1, 1)-cell by setting $X^{1,1}_7 = 1$ and $X^{1,1}_k = 0$ for ($k = 1,\ldots ,6,8,9$). Conversely, imposing the condition $X^{1,1}_7 = 0$ lets us explicitly exclude 7 from (1, 1)-cell. This naturally inspires us to specifically reject the *k*-th item from the *i*-th row of the $P \times Q$ grid by introducing multiple constraints, i.e., $X^{i,j}_k = 0$ for $j=1,\ldots ,Q$. This formulation can be applied when we need to align items of a specific size in a column or a row. Thus, if we know the sizes of items beforehand, we can intentionally align items of the same size in a specific row or column. Figure 3 presents such an example, where we successfully restrict small cans to appear in the bottom row only by rejecting cans of other sizes.

*(S2) Placing two items of the same category next to each other*

The layout rule (S2) plays an essential role in promoting users’ favorite items. This is because they are likely to identify what kinds of items are present in the layout at first glance and identify their most preferred items in the neighborhood of relevant ones. This design principle also inspires them to explore their favorites even when they have not yet decided what they really want. We often respect this convention for arranging items to intentionally draw more attention from viewers. Examples include arrangements of products on supermarket shelves, where we are instructed to place items in the same category next to each other.

Let us explain our formulation with the $P \times Q$ grid case, where we intended to place the two numbers *k* and *l* so that they are horizontally adjacent to each other. One simple case is to place *k*-th and *l*-th items in (*i*, *j*)- and $(i,j+1)$-cells, respectively, which results in equation $X^{i,j}_k + X^{i,j+1}_l = 2$. We can rewrite this constraint by introducing an additional binary variable, $\chi ^{i,j}_{k,l}$, as:

1 $$\begin{aligned} 2 - G(1 - \chi ^{i,j}_{k,l}) \le X^{i,j}_k + X^{i,j+1}_l \le 2 + G(1 - \chi ^{i,j}_{k,l}), \end{aligned}$$

where $G$ represents a large constant value and is set to be 128 by default in our implementation. This condition holds if $\chi ^{i,j}_{k,l} = 1$. Of course, we can exchange the positions of *k* and *l*. We can implement this by introducing another binary variable, $\chi ^{i,j}_{l,k}$, and imposing a similar condition as Eq. (1). If we do not care about the order of the two items in (*i*, *j*)- and $(i,j+1)$-cells, we can write the condition as $\chi ^{i,j}_{k,l}+\chi ^{i,j}_{l,k}=1$. Since our ultimate goal is to place the two numbers next to each other in a row, we can write the condition by enumerating all possible cases in the $P \times Q$ grid, as follows:

$$\begin{aligned} \sum _{i=1}^P \sum _{j=1}^{Q-1} ( \chi ^{i,j}_{k,l} + \chi ^{i,j}_{l,k} ) = 1. \nonumber \end{aligned}$$

In our implementation, we encoded this type of requirement as a soft constraint by summing up $w\bigl ( \sum _{i=1}^P \sum _{j=1}^{Q-1} ( \chi ^{i,j}_{k,l} + \chi ^{i,j}_{l,k} ) \bigr )$ to the overall objective function to be maximized. $w$ indicates a specific weight value for this constraint and can be adjusted according to the degree of requirement. A similar formulation can be employed to align two items next to each other in a column. Figure 3a shows an example in which drinks of the same category are placed next to each other. In practice, we can find juice bottles, black tea bottles, and small coffee cans next to each other in a row, while oolong tea bottles and cans are aligned vertically adjacent to each other.

*(S3) Arranging the same items in a sub-matrix*

The placement guideline (S3) successfully prevents the user from being distracted by identical items repeatedly scattered separately over the display. It is also better to place important items in a block to draw the user’s attention using a proposed layout strategy that is aesthetically pleasing. The most common scenario associated with this guideline is to line up the same items in multiple cells in a group. Suppose that we want to line up the *k*-th item *m* times in a row in the $P \times Q$ grid. Here, we can identify a set of possible sequences of *m* cells as (*i*, *j*)-, $\cdots$, and $(i,j+m-1)$-cells where $i = 1,\ldots ,P$ and $j = 1,\ldots ,Q-m+1$. This observation leads us to the formulation for the sequence of cells as

$$\begin{aligned} m - G(1 - \chi ^{i,j}_{\underbrace{k,\ldots ,k}_{{m}\;\text{ times }}}) \le \sum _{\delta=1}^m X^{i,j+{\delta-1}}_k \le m + G(1 - \chi ^{i,j}_{\underbrace{k,\ldots ,k}_{{m}\;\text{ times }}}), \nonumber \end{aligned}$$

again by introducing a new binary variable, $\chi ^{i,j}_{\underbrace{k,\ldots ,k}_{{m}\;\text{ times }}}$. In our formulation, we simultaneously impose a hard constraint to prohibit the sequence of the *k*-th items from splitting into multiple separate groups by imposing the following constraints:

$$\begin{aligned} \sum _{i=1}^{P} \sum _{j=1}^Q X^{i,j}_k = m \quad \text{ and } \quad \sum _{i=1}^P \sum _{j=1}^{Q-m+1} \chi ^{i,j}_{\underbrace{k,\ldots ,k}_{{m}\;\text{ times }}} = 1. \nonumber \end{aligned}$$

Fig. 3b exhibits an example of three mineral bottles lined up in a row. This can be easily extended to arrange the same item in an $n \times m$ sub-matrix in the $P \times Q$ grid by introducing the constraints

$$\begin{aligned} \sum _{i=1}^{P} \sum _{j=1}^Q X^{i,j}_k = n \times m \quad \text{ and } \quad \sum _{i=1}^{P-n+1} \sum _{j=1}^{Q-m+1} \chi ^{i,j}_{\underbrace{k,\ldots ,k}_{{n \times m}\;\text{ times }}} = 1, \nonumber \end{aligned}$$

where

2 $$\begin{aligned} n \times m - G(1 - \chi ^{i,j}_{\underbrace{k,\ldots ,k}_{{n \times m}\;\text{ times }}}) \le \sum _{\gamma=1}^n \sum _{\delta=1}^m X^{i+\gamma-1,j+{\delta-1}}_k \le n \times m + G(1 - \chi ^{i,j}_{\underbrace{k,\ldots ,k}_{{n \times m}\;\text{ times }}}). \end{aligned}$$

See Fig. 7 for placements of items in sub-matrix forms, where each block of images is replaced with a larger image.

## Gaze-driven dynamic placement

Improving the static placement of items is essential to draw attention to specific items, especially for print media, such as newspapers and magazines. However, contemporary digital signage technologies promote superior demonstration of items by dynamically updating the selection and arrangement of items on display devices. This is crucial because we need to guide item selection for users by customizing item placement according to the intermediate context of searching for their favorite items. In our research, we employ an eye-tracking device to understand the current focus on the items by analyzing the spatiotemporal distribution of the visual attention of viewers. We then adaptively rearrange the layout of items to maximally respect the ongoing interest in searching for their preferred items. In this section, we explain a technique for retrieving the temporal change in the spatial distribution of visual attention on the display and updating the associated placement of items accordingly. In the next section, we will detail our approach for replacing visible items with those hidden behind the display as well as rearranging them by referring to the ongoing search context.

### Computing the distribution of visual attention

An eye-tracker assists our investigation of the spatiotemporal movement of eye gaze points to understand how viewers focus on specific areas of interest in a scene. In our approach, we make maximum use of the eye-tracker for identifying which subset of items from the visible item set draws the most visual attention. This scheme allows us to improve the placement of items based on the distribution of such gaze points.

In practical scenarios, we compute the spatiotemporal distribution of visual attention by convolving each gaze point in the sequence with a Gaussian weighting kernel (Daae Lampe and Hauser 2011). We often visualize the spatial distribution of visual attention as a colored map called a *heatmap*, in which the color changes from blue to green to red as the degree of attention increases. Figure 1a shows the placement of drinks with the overlaid heatmap, where cola bottles around the top right corner attracted the most visual attention of the viewer. Moreover, we visualize the spatiotemporal trajectory of the eye gazes as gaze plots in the figure, where the gaze point is represented as larger if it was a more recent one. In our heatmap computation, we incorporate gaze points recorded within the last two seconds, and the Gaussian weight is scaled down linearly in terms of the time from the present.

### Updating the priority values of items

In our problem setup, our challenge is to replace the items on display and rearrange them according to the preferences of viewers by analyzing their ongoing context while searching for their favorites. This means that we want to maximize their satisfaction by presenting their favorite items on display and hiding unwanted ones through dynamic rearrangement. This is accomplished by maximizing the objective function that includes the weighted sum of binary variables assigned to the following items

3 $$\begin{aligned} \sum _{k=1}^N \alpha _k (\sum _{i=1}^P \sum _{j=1}^Q X^{i,j}_k ), \end{aligned}$$

where $\alpha _k \, (k = 1,\ldots ,N)$ is the priority value of the *k*-th item. Here, we assume that each priority value $\alpha _k$ is normalized into [0, 1]. We can increase Eq. (3) as we accommodate more items associated with higher priority values in the grid. This implies that items having low priority values are likely to be excluded from the current list of candidate items for selection and thus become invisible. Specifically, the number of cells dominated by the *k*-th item depends on the choice of its priority value $\alpha _k$ and the limits for the number of its appearances $[L_k, U_k]$ mentioned in Sect. 3.1. In our scenario, we expect that items that are initially hidden behind the display will replace visible items when the corresponding priority values become higher. Thus, our task here is to formulate a scheme for plausibly updating the priority values $\alpha _k \, (k= 1,\ldots ,N)$ that reflect the individual preferences of the items.

For this purpose, we update the priority value of each item by calculating the integral of the heatmap within the area of each cell in the grid. The integrated values are accumulated for each item if the corresponding item dominates multiple cells. Suppose that we obtain the percentage of the entire visual attention for the *k*-th item as $r_k$. In our implementation, we replace $r_k$ with an enhanced version, $r^{\prime }_k$, by applying a *winner-takes-all* enhancement to the distribution of the percentage values. This is biologically inspired by the concept of a *saliency map* (Itti et al. 1998; Koch and Ullman 1987), since we are likely to focus on a unique region of interest that pops up the most from the scene. Finally, we linearly normalize $r^{\prime }_k \, (k=1,\ldots ,N)$ so that the maximum value of $r^{\prime }_k \, (k=1,\ldots ,N)$ becomes 1.0. These normalized rates of visual attention guide us to find the updated priority values for $\alpha _k \, (k = 1,\ldots ,N)$.

### Design criteria for dynamic placement

We employ the following criteria for controlling the dynamic placement of items.

1. Fixing primarily focused items to their corresponding cells
2. Respecting the most focused item to prepare the subsequent placement

These two criteria serve as the basis for further elaborating the dynamic updating of item selection and placement, which can be detailed below.

*(D1) Fixing primarily focused items to their corresponding cells*

As the first design criteria for dynamic placement, we fix the positions of items that attract more attention to prevent viewers from being surprised to find them suddenly jumping out of their field of view. This is implemented by adding temporary constraints to fix the items at the corresponding cells when computing the subsequent placement. To select such a set of focused items, we first sum up the integrals of the heatmap within the cells associated with the items. We then identify each of them as a focused item if the corresponding sum is more than the predefined threshold. In our implementation, one to three different kinds of items are identified as specifically focused items. This design specification allows viewers to freely focus on their favorite items without being disturbed by other items that will potentially be replaced later.

*(D2) Respecting the most focused item to prepare the subsequent placement*

Of course, we need to respect how the viewers pay visual attention to the items on display and maximally consider their choices for preparing the following layout. As described earlier, we compute the degree of visual attention for each item by summing up the heatmap values over the corresponding cells, and we identify the most focused item. By adjusting the priority value associated with the most focused item, we can present this item together with the closely related ones in the subsequent display for the convenience of the viewers.

One may feel that handling the most popular item as a particular one could cause an unexpected problem, especially when the first and second top items attract an almost equivalent amount of attention. Nonetheless, such competing items are often likely to be close to each other in the grid placement. This is due to the characteristic in the spatial distribution of the heatmap within a relatively short period. Thus, such items are often in the same category due to the design criterion (S2) employed in the static placement. This indicates that selecting the most attractive item or others close to it does not significantly affect the subsequent placements of items. A major exception will be discussed in Sect. 6.3.

## Context-aware updates of items

Our formulation for updating the priority values does not explicitly consider items hidden behind the display. This inspires us to increase the priority values of such invisible items so that they can replace visible ones that are not of interest. For this reason, we want to adequately understand the ongoing context in which the viewers try to find their favorite items. Our solution here is to construct a *context map* that retains plausible association rules among items in diagram style. This means that the map represents pairwise relationships between the items as a spatial layout of the corresponding points on a 2D diagram. The association rules enable us to instantly discern the preferred items to be exhibited next in the display from the most focused items at present. This becomes possible by retrieving the nearest neighbors within the vicinity of the item currently in focus on the map. Successively following the association rules helps us adaptively adjust the priority values of the items according to the history of the most focused item, which is identified by faithfully interpreting the distribution of the viewers’ visual attention.

### Topic-based mining of annotated texts

It is often the case that the automatic construction of context maps requires considerable effort to reproduce the authentic relationships among items. Undoubtedly, we can take advantage of machine learning techniques if we have easy access to extensive training datasets that reveal how specific items are selected simultaneously. However, we need to provide alternatives because such training data are not immediately available when presenting items to newcomers through display devices.

Our choice here is to employ text mining techniques that infer meaningful relationships between pairs of items by taking as input associated annotated texts. It is often effortless to collect explanatory texts about the individual items, such as from internet resources. In particular, we introduce topic-based text analysis to efficiently reproduce association rules among them.

Topic modeling (Blei 2012) is a technique for extracting important topics and has been developed in the area of natural language processing. We can characterize the explanatory description for each item as the linear sum of such representative topics, each of which constitutes a weighted sum of basic terms. Suppose that the *i*-th topic is represented as follows:

$$\begin{aligned} \tau ^{i} = \beta ^{i}_{1} \omega ^{i}_{1} + \beta ^{i}_{2} \omega ^{i}_{2} + \ldots , \qquad (i = 1,\ldots ,R), \end{aligned}$$

where $\{\omega ^{i}_r\} \, (r = 1,2,\ldots )$ are the basic terms that constitute the *i*-th topic, $\{\beta ^{i}_r\}$ are the associated weight coefficients, and $R$ is the total number of topics. Here, $R$ is optimized in a pre-process by finding the best number that maximizes a coherence score during the topic analysis. We specifically focus on nouns as our target terms and excluded other categories of terms such as verbs, adjectives, adverbs, and prepositions. This effectively allows us to extract relationships among items since the nouns refer to names and identities commonly shared by items of a specific category.

Once we can extract the specific number of topics $\{\tau ^{i}\} \, (i = 1,\ldots ,R)$, we transform the annotated text associated with the *k*-item as an $R$-dimensional feature vector, consisting of $R$ coefficients assigned to the respective topics, as

$$\begin{aligned} (\lambda ^{1}_k, \ldots , \lambda ^{R}_k), \qquad (k = 1, \ldots , N). \nonumber \end{aligned}$$

This implies that we represent each item as a feature vector consisting of such coefficients, which allows us to measure the similarity between every pair of items by computing the index of dissimilarity between the corresponding feature vectors. In our approach, we project an $R$-dimensional feature vector of each explanatory text onto the 2D space using t-SNE (van der Maaten and Hinton 2008) to construct the context map. The map actually helps us search for a specific number of items related to the one most focused on by referring to their spatial positions in its 2D domain. In our approach, we employ this 2D projected map not only for visualization purposes but also to retrieve items that are closely relevant to the current most focused item. Refer to Sect. 5.2 for further details.

**Fig. 4:**
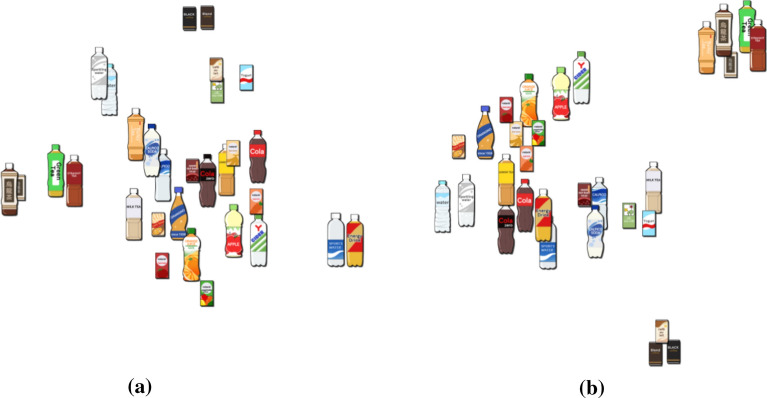
A context map obtained using topic analysis based on **a** LDA and **b** NMF. The number of topics is 12 in both cases. The NMF-based analysis better elucidates the configuration among drink categories

LDA (Blei et al. 2003) is one of the most widely used techniques in topic modeling. We have previously employed LDA for that purpose (Takahashi et al. 2020); however, as pointed out by Kim et al. (2015), LDA is not scalable enough to handle even medium-sized collections of texts and often requires relatively long computation times. In this study, instead of LDA, we employed NMF as our topic modeling method by referring to the matrix spanned by occurrence histograms of the representative words for the respective documents. We employed *time frequency-inverse document frequency* (TF-IDF) (Robertson 2004) as a weighting factor to transform the word occurrence matrices before factorizing them in order to properly consider the importance of the respective words in the overall document set. We also found that the NMF-based topic extraction was likely to discriminate between drink categories as faithfully as expected. Figure 4 shows such a comparison between the 2D context maps obtained using LDA and NMF.

### Design criteria for context-aware priority updates

As described earlier, our dynamic placement was implemented by adjusting the priority values $\alpha _k \, (0< \alpha _k < 1, k = 1,\ldots ,N)$ associated with the given *N* items. The context map provides us with association rules among the items so that we can select the items that will attract the viewers’ attention next. In connection with the association rules based on the context map, we introduce two additional criteria for the design of dynamic placement:

(D3) Proactively replacing visible items with hidden ones
(D4) Prioritizing items closely associated with the one currently in focus

These design criteria permit us to replace the current priority value of the *k*-th item with the new value after it is normalized. We describe our implementation of these design criteria that present preferred items to viewers.

*(D3) Proactively replacing visible items with hidden ones*

In our approach, we proactively replace unwanted visible items with those hidden behind the display because they are more likely to attract the visual attention of the viewers. Another reason is that we want to make the best use of time by presenting as many items as possible within a fixed period. Thus, we update the priority values of items currently visible on the display according to the degree of interest they attract. We also increase the priority values of invisible items without exception to intentionally replace unwanted visible ones in the next update. This strategy will proactively replace current items with invisible ones that can potentially attract more visual attention.

However, we still run the potential risk of missing some items of interest if the corresponding priority values are kept relatively low during a specific period of time. This may be inevitable even though we try to maximally bring hidden items into the display by raising their priority. We will discuss this issue later in Sect. 5.3.

*(D4) Prioritizing items closely associated with the one currently in focus*

At the same time, we faithfully follow association rules yielded by the context map to select the following items to be exhibited. More specifically, we activate items that have a strong association with the most focused item by increasing their priority values, regardless of whether they are currently on display or not. For this purpose, we conduct the *k*-nearest neighbor search around the currently attended item on the context map by calculating the 2D distance from it.

Another option is to retrieve the similarity between items by directly computing mutual distances in the original high-dimensional feature space. In our approach, we did not employ this because we may accidentally include irrelevant items in the list of neighbors. In practice, topic-based text mining successfully identifies a group of closely related items as a cluster in the feature space. However, it is still possible that such clusters are intricately embedded in the original high-dimensional space, and items belonging to different clusters happen to be close to each other unexpectedly. This is because each item has a high-dimensional neighborhood, which means that many other items can easily approach it from any direction. The dimensionality reduction based on t-SNE successfully retains the mutual closeness of items in each cluster while keeping different clusters apart. This allows us to successfully retrieve a set of closely related items to the currently focused one from the 2D projected version of the context map.

We actually tested the approximate nearest neighbor search (Li et al. 2019) on the high-dimensional context map before applying dimensionality reduction (i.e., t-SNE). However, in this case, we encountered several undesirable cases in which we retrieved irrelevant items. Thus, we decided to employ the configuration of the items after they are projected onto 2D space.

### Notes on the initial placement of items

As described previously, the design criterion (D3) may risk hiding a particular set of items if their priority values are kept lower than those of other items. We can considerably mitigate this risk with the help of the proposed context-aware adjustment of the priority values for the items. However, it may be possible that items of some specific category remain invisible on the display if their priority values are not fully activated by their related items through the association rules on the context map.

This issue can be alleviated by guiding the initial placement so that the visible items represent their different categories. The context map also facilitates the selection of such initial items since the clusters of items on the map effectively characterize such categorization. In practice, in our implementation, we simulate a conventional layout rule that picks up representative items from different categories by raising their initial priority values intentionally. This means that following this rule is equivalent to choosing important items from each cluster on the context map. In this way, devising the initial placement of items allows users to avoid missing their preferred items even when they belong to any category.

## Results

In this section, we first demonstrate the experimental results of our approach, then we justify the design criteria for the context-aware placement of items through user studies, and finally, we discuss the feasibility of our formulation.

We implemented our prototype system on a MacBook Pro with an Intel Core i7 processor (four cores, 2.3GHz, 512KB L2 Cache per core, and 8MB L3 Cache) and 32GB of RAM. The source code was written in C++ using OpenGL/GLUT for UI and drawing items, OpenCV for handling images, and IBM ILOG CPLEX for solving IP optimization problems. Python programs were also combined with the system to conduct a topic-based analysis of annotated texts based on NMF. We introduced a Tobii Pro Nano eye-tracker together with the Tobii SDK library to retrieve the viewer’s gaze positions. The annotated texts associated with the items were collected from Internet resources, such as Wikipedia.

**Fig. 5:**
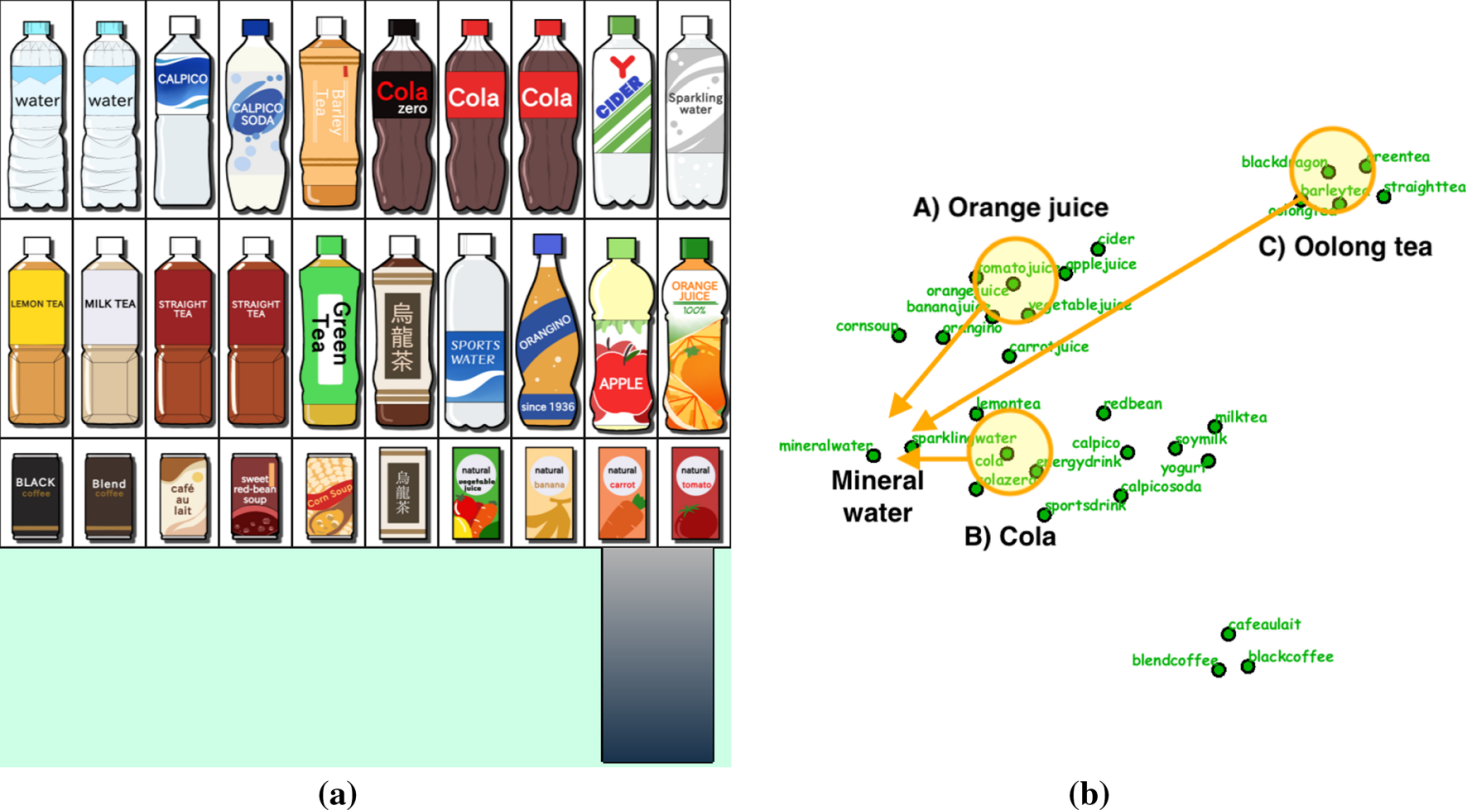
Three scenarios for exploring drinks with a virtual vending machine.** a** The initial grid placement of drinks.** b** The context map with three scenarios for exploring drinks

**Fig. 6:**
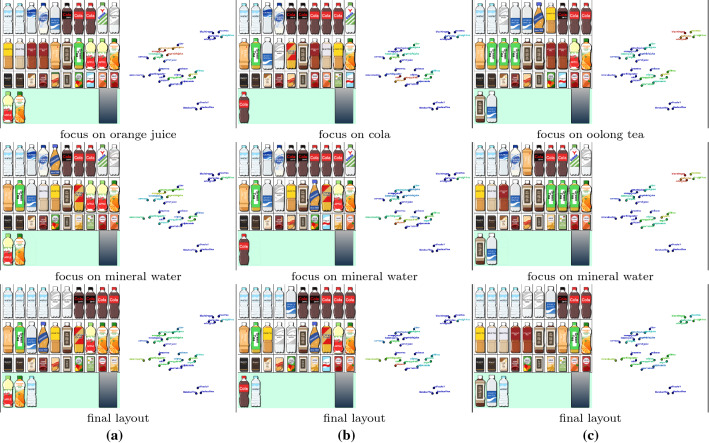
Changes in the placement of drinks according to the three scenarios (from top to bottom in each column). **a** From orange juice to mineral water. **b** From cola to mineral water. **c** From oolong tea to mineral water

**Fig. 7:**
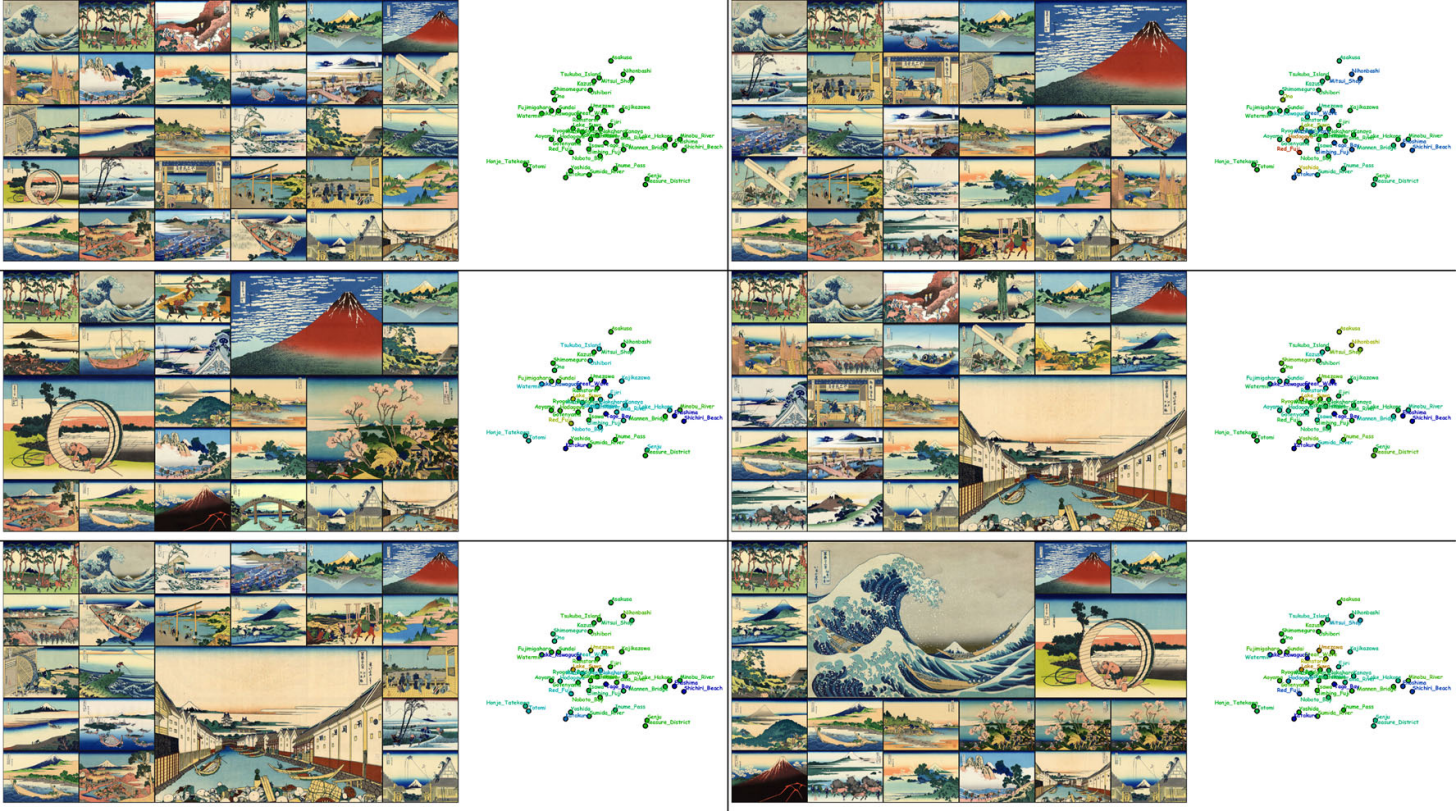
System snapshots exploring landscape prints in the series of paintings, *Thirty-Six Views of Mt. Fuji* (from left to right and top to bottom). Each slot consists of the grid placement of images (left) and the context map (right)

### Experimental results

In the first demonstration of our approach, we constructed a virtual vending machine of drinks, as exhibited in Fig. 1. We composed three search scenarios to see how the context-aware approach produces different layouts of drinks, as follows: A) from orange juice to mineral water, B) from cola to mineral water, and C) from oolong tea to mineral water. Figure 5a and b present the initial placement of drinks and the corresponding context map computed by topic-based text mining using matrix factorization. We also clarify how our search path can be indicated on the context map according to the three scenarios in Fig. 5b.

In the initial placement, we first classified the set of drinks into several categories. We then employed the design criterion (S2) to place drinks from similar categories next to each other, including water (mineral and sparkling), cultured milk (Calpico, etc.), tea (black, green, oolong, etc.), and juice (orange, apple, etc.). Drinks of different sizes are arranged in a vertical alignment (i.e., a bottle and a can of oolong tea). We use the criterion (S1) to restrict small cans and brick cartons to the bottom row. We force bottles with the same drink to line up horizontally with the criterion (S3). In this case, we also bound the numbers of mineral water, sparkling water, cola, cola zero, orange juice, apple juice, oolong tea, and green tea bottles within [2, 4], [1, 2], [2, 3], [1, 2], [1, 2], [0, 2], [1, 2], and [1, 3].

The critical mechanism is that the system waits for the visual attention of viewers to be entirely focused on a small area of the display before updating the placement of items. The system also lists items attracting more attention in the *hotlist* to record the history of preferred items in the bottom row and next to the output port of the vending machine (Fig. 5a). We smoothly replace items in other cells using a cross-dissolve animation in order not to disturb the viewers’ focus on their preferred items.

Figure 6 presents sequences of snapshots to depict how the three scenarios control the dynamic placement of drinks differently. The context map represents the priority value of each drink by coloring its corresponding plots and name labels, so that the color changes from blue to yellow to red as the priority value increases. In Scenario A), we begin by focusing on orange juice in the middle row on the right and replace unattended items with an additional apple juice bottle by following the association rules provided by the context map. Although the layout still kept multiple juice bottles, it soon added mineral water along with sparkling water once the mineral water attracted more attention. In Scenario B), more cola and cola zero bottles were lined up in the first half and still stayed on display even after mineral water bottles attracted more attention. The same effect can be found in Scenario C), where oolong tea, along with other tea bottles, joined the placement. Mineral water bottles finally won more cells on the display, while most of the tea bottles remained in the end. These results demonstrated that we could successfully take advantage of the association rules based on the context map to maximally respect the underlying context of searching for favorite items.

We also simulated the digital information wall in which we arranged a series of landscape woodblock prints called *Thirty-Six Views of Mt. Fuji* painted by the famous Japanese Ukiyo-e artist, Hokusai Katsushika. Figure 7 demonstrates how the paintings are updated dynamically according to the spatiotemporal distribution of visual attention. We supposed a possible scenario in which visitors in a museum freely looked at their favorite paintings on the information wall and simulated how the system dynamically rearranged the paintings when they paid particular attention to the display. We again employed the context map obtained using topic modeling based on matrix factorization to prepare a set of association rules among the paintings. In this setup, we grouped multiple cells into a single block matrix if they retained the same painting.

### User evaluation studies

We recruited participants for user studies and asked them to evaluate the validity of each of the design criteria (S1)–(S3) and (D1)–(D4), which were described earlier in this paper. In the user studies, we evaluated each of the design criteria by invalidating each criterion to generate the different placement of items and asked the participants to conduct a side-by-side comparison with the original placement obtained with the complete set of criteria. For the respective criteria for designing the static placement (S1)–(S3), we asked each participant to complete an online questionnaire. We conducted an eye-tracking study to validate the design criteria for the dynamic placement (D1)–(D4). We also compared the association rules suggested by our context map with those obtained with a conventional recommendation system based on matrix factorization.

#### Justifying the design criteria (S1)–(S3)

**Fig. 8:**
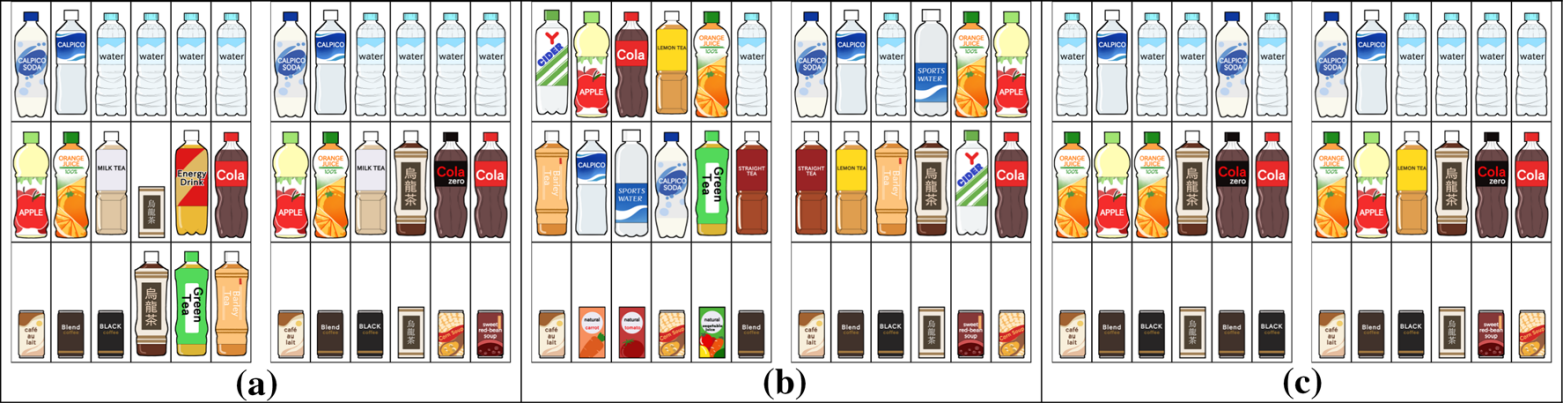
Static placements of drinks used in the user study. The $3 \times 6$ placements were optimized (**a**) without (S1) (left) and with (S1) (right), (**b**) without (S2) (left) and with (S2) (right), and (**c**) without (S3) (left) and with (S3) (right). The two placements for each comparison differed due to the change in the selection of design criteria and their associated constraints. Other parameters, including the initial priority value and upper and lower limits on the number of appearances for each item, were the same

**Fig. 9:**
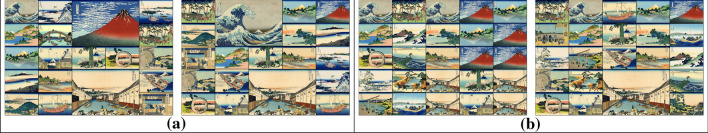
Static placements of paintings used in the user study. The $5 \times 5$ placements were optimized (**a**) without (S2) (left) and with (S2) (right), and (**b**) without (S3) (left) and with (S3) (right). The two placements for each comparison differed due to the change in the selection of design criteria and their associated constraints. Other parameters, including the initial priority value and upper and lower limit on the number of appearances for each item, were the same

**Table 1:** Side-by-side comparisons between static placements for the criteria (S1)–(S3). Also refer to Figs. 8 and 9

Design criteria		Without criterion		With criterion		*p*-value
(S1) in the $3 \times 6$ placement of drinks		6 (13.6%)		38 (86.4%)		$4.72 \times 10^{-7}$
(S2) in the $3 \times 6$ placement of drinks		11 (25.0%)		33 (75.0%)		$6.30 \times 10^{-4}$
(S3) in the $3 \times 6$ placement of drinks		6 (13.6%)		38 (86.4%)		$4.72 \times 10^{-7}$
(S1) in the $2 \times 10$ placement of drinks		4 (9.1%)		40 (90.9%)		$8.53 \times 10^{-9}$
(S2) in the $2 \times 10$ placement of drinks		5 (11.4%)		39 (88.6%)		$7.03 \times 10^{-8}$
(S3) in the $2 \times 10$ placement of drinks		9 (20.5%)		35 (79.5%)		$5.30 \times 10^{-5}$
(S2) in the $5 \times 5$ placement of paintings		17 (38.6%)		27 (61.4%)		$8.71 \times 10^{-2} (> 0.05)$
(S3) in the $5 \times 5$ placement of paintings		4 (9.1%)		40 (90.9%)		$8.53 \times 10^{-9}$

We initiated our user evaluation by assessing the design criteria (S1)–(S3) for the static placement of items through an online questionnaire. We first prepared three pairs of drink placements in $3 \times 6$ and $2 \times 10$ grids, in each of which we excluded one of the three design criteria (S1)–(S3) for the comparison by the participants. We also prepared $5 \times 5$ grid layouts of paintings; in this case, we tested the design criteria (S2) and (S3) only because all of the paintings are in the same size and aspect ratio. We recruited 44 participants (8 females and 36 males) for the online questionnaire. Their ages ranged from 19 to 65, and more than half were university students (ages 19–24) majoring in computer sciences and relevant fields. For each evaluation task, we requested the participants to conduct side-by-side comparisons between a pair of static placements described above and select the one they liked. We provided participants with information about the aspect in which the two placements were different without informing them about which placement misses the corresponding design principle.

Table 1 exhibits the comparison results we collected from the online questionnaire. Figure 8 lists the first three pairs of drink layouts, and Fig. 9 presents the last two pairs of painting placements used in this study. In the first pair in Fig. 8a, drinks of small size are scattered over the arrangement on the left due to the absence of (S1), while they are all aligned in the bottom row on the right. Thus, most of the participants indicated the placement on the right as their favorite choice. Figure 8b presents the side-by-side comparison between the placements of drinks while the left placement lacks the design criterion (S2). As shown in the layout on the right, drinks in the same category are arranged next to each other, including juice bottles, tea bottles, and carbonated drink bottles. On the other hand, tea bottles are irregularly located, and juice bottles are split in the arrangement on the left. The majority of the participants supported the placement on the right also in this case. The last design criterion, (S3), was employed to systematically line up multiple bottles of mineral water, as exhibited on the right, while they were split into multiple blocks on the left. Again, in this case, the design criteria were liked by many participants. Table 1 also demonstrates that these three design criteria collected the majority of votes even in a $2 \times 10$ grid placement of items.

As for the placement of paintings in Fig. 9a, the design criterion (S2) was only weakly supported. This was probably because fewer participants noticed that the paintings were grouped individually according to the target motifs, such as mountains, seas, ships, and streets. Conversely, the last rule (S3) was explicitly supported because copies of the same painting were aesthetically grouped in a block, as presented in Fig. 9b. In summary, all the three design criteria (S1)–(S3) in both cases were highly evaluated in the user study to varying degrees. We will discuss this issue again later in this section.

#### Justifying the design criteria (D1)–(D4)

Evaluating the design criteria in the context of the dynamic placement of items requires us to carefully design an eye-tracking study as a laboratory experiment. For this purpose, we prepared a scenario that guides each participant to watch a specific set of items in a specific order. We then screencast the actual update of the dynamic placement so that the participants could refer to how the items were replaced and rearranged according to their spatiotemporal eye gaze movements.

**Fig. 10:**
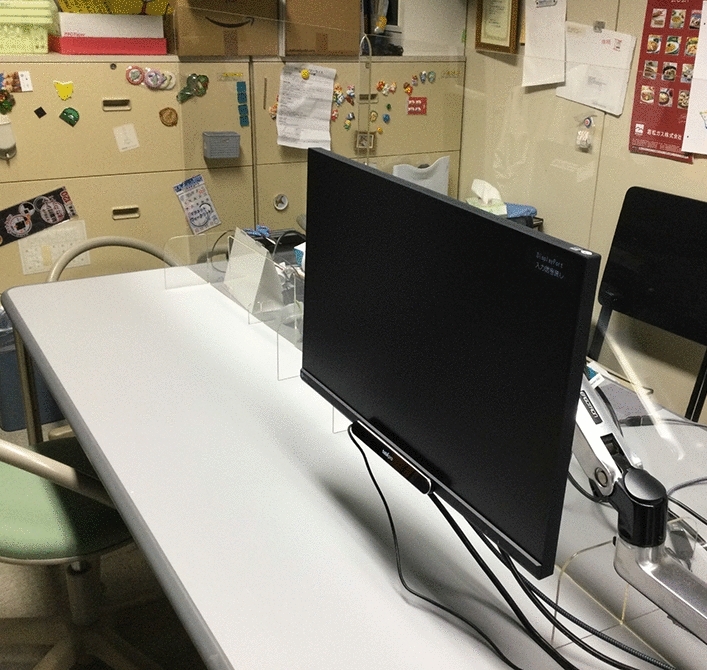
Laboratory space for conducting the eye-tracking study

We recruited eight participants with normal vision; all participants were male university students majoring in computer sciences, and their ages ranged from 19 to 24. As our running example, we employed the dynamic placement of drinks. We performed an approximately 30–40 minute study for each participant, which consisted of explaining the objectives and overall scenario of the experiment, obtaining a written approval form, calibrating the eye-tracking devices, and performing a preliminary practice followed by four comparison sessions. Each session contained a comparison between a pair of dynamic placement of items, where the participants were guided to look at cola (bottle), oolong tea (bottle), and orange juice (bottle), in that order, until the overall placement of drinks was updated twice for each drink. In each section, we asked the participants to select the better dynamic presentation of drinks while invalidating one of the design criteria (D1)–(D4) in either of the two eye-tracking tasks. Although we informed the participants of how the two placements differed in advance, we did not tell them which placement corresponded to the case in which we invalidated the criterion. This experiment was conducted under the approval of the research ethics committee, and adequate measures against COVID-19 were taken to protect the participants (See Fig. 10). In particular, in this eye-tracking study, each participant was compensated with 1000 JPY because they needed to spend longer time for their participation.

**Fig. 11:**
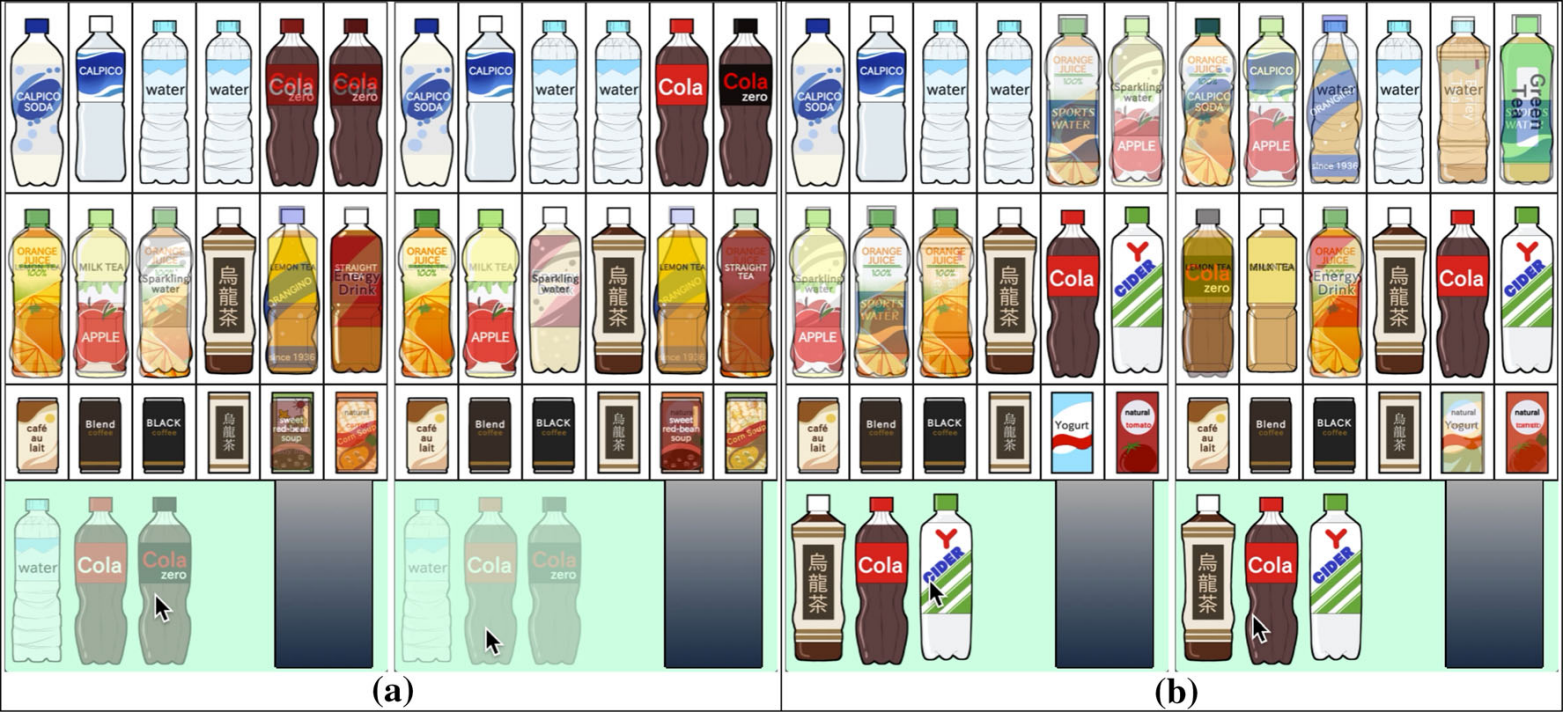
The $3 \times 6$ dynamic placement of drinks in the user study when the cola bottle is the most focused. The layouts are designed **a** without (D1) and with (D1), and ** b** without (D4) and with (D4)

Table 2 presents the comparison results of this laboratory experiment. Again, all the design criteria were supported highly by the participants. In the comparison task for the design criterion (D1), all the participants liked the focused item to stay in the same cell for a while, as shown in Fig. 11a. In the second comparison for the criterion (D2), most participants rejected the dynamic placement in which we prepared the next update by ignoring the drink that attracted the most visual attention. The participants preferred the criterion (D3) that exhibited various drinks, including those hidden behind the display. Lastly, our context-aware dynamic placement criterion (D4) received higher ratings from many of the participants. The rightmost image in Fig. 11b shows such a case, in which the cola zero bottle and matched carbonated drink bottles were more likely to appear on the display after the participants focused their attention on the cola bottle, especially when it is compared with the case on the left. These observations demonstrate that our design criteria for dynamic placement promote an attractive exhibition of items according to the visual interest of the viewers.

**Table 2:** Side-by-side comparisons between dynamic placements for the criteria (D1)–(D4)

Design criteria		Without criterion		With criterion		*p*-value
(D1) in the $3 \times 6$ placement of drinks		0 (0.0%)		8 (100.0%)		$3.91 \times 10^{-3}$
(D2) in the $3 \times 6$ placement of drinks		2 (25.0%)		6 (75.0%)		$1.45 \times 10^{-1} (> 0.05)$
(D3) in the $3 \times 6$ placement of drinks		1 (12.5%)		7 (87.5%)		$3.52 \times 10^{-2}$
(D4) in the $3 \times 6$ placement of drinks		1 (12.5%)		7 (87.5%)		$3.52 \times 10^{-2}$

#### Comparison with conventional association rules

**Table 3:** Side-by-side comparisons between recommended drinks using the matrix factorization and context map. The percentage to the right of each recommended drink represents the approval rating in the user study

Focused item	Matrix factorization		Context map	
Cola	Energy drink (73.0%)		Energy drink (73.0%)	
	Cola zero (94.6%)		Cola zero (94.6%)	
	Sparkling water (40.5%)		Lemon black tea (0.0%)	
Oolong tea	Green tea (75.7%)		Green tea (75.7%)	
	Barley tea (86.5%)		Barley tea (86.5%)	
	Lemon black tea (13.5%)		Black tea (43.2%)	
Orange juice	Banana juice (56.8%)		Vegetable juice (51.4%)	
	Oolong tea (0.0%)		Tomato juice (35.1%)	
	Soy milk (0.0%)		Banana juice (56.8%)	
Blend coffee	Black coffee (91.9%)		Black coffee (91.9%)	
	Cafe au lait (94.6%)		Cafe au lait (94.6%)	
	Green tea (2.7%)		Cultured milk soda (0.0%)	
Mineral water	Sparkling water (94.6%)		Sparkling water (94.6%)	
	Soy milk (0.0%)		Cola zero (0.0%)	
	Cola zero (0.0%)		Lemon black tea (8.1%)	

For the final user study, we compared association rules among items based on our context map with those obtained by conventional recommendation techniques. For this purpose, we again employed the association rules among drinks as an example. To retrieve the association rules reproduced by the recommendation techniques, we first recruited 30 participants (7 females and 23 males; ages ranged from 19 to 59) who majored in computer sciences and related fields, and asked them to complete an online questionnaire. In the questionnaire, they were requested to answer five questions, including “Which drinks do you like when you are thirsty, tired, etc.,” and they were asked to select a specific set of drinks from 30 available drinks for each question. From this data, we composed a $30 \times 150$ matrix, where the matrix is spanned by 150 ($=$ 30 persons $\times$ 5 questions) binary vectors representing how the 30 drinks were selected simultaneously for the individual cases. We applied NMF (Koren et al. 2009) to decompose the matrix into representative patterns of drink selections and inferred which drinks should be recommended if one specific drink was chosen. The results are listed in Table 3, which shows the three relevant drinks recommended by the two approaches if we visually focus on a specific drink. As for the approval ratings for the respective drinks in Table 3, we administered another online questionnaire to which we invited 37 participants (7 females and 30 males; ages ranged from 19 to 65) to answer whether they approved of the recommendation for each candidate drink.

Comparing the recommended drinks enables us to claim that our context map can produce drink association rules as reasonable as those obtained by conventional recommendation techniques. These results also support the validity of our context-aware dynamic placement of items.

### Discussion

As described above, we could confirm that our design choices for static and dynamic placement of items were largely supported by the participants in the user studies. To verify this claim, we computed *p*-values for each side-by-side comparison between the two placements on the assumption that the probability of choosing either of them is 0.5 (50%) for both. Tables 1 and 2 list the *p*-values for each comparison. If we employ 0.05 (5%) as a threshold for the significance test, all the biases in the layout selections were statistically significant, except for (S2) in the $5 \times 5$ grid placement of paintings and (D2) in the eye-tracking experiment. Even in these exceptional cases, the *p*-values were relatively close to the threshold for statistically significant biases. This consideration lets us conclude that we can statistically support the proposed design criteria for static and dynamic placement.

The proposed design criteria for the dynamic placement of items have limitations and remain to be further elaborated. In the design criterion (D2), we hypothesize that viewers focus on a set of items of the same category in the search for their favorites. However, they may try to intentionally compare items of different categories, especially in the early stage of their visual exploration. In this exceptional case, our system expects viewers to focus on some item first and then shift to another item of a different category. The proposed approach tries to faithfully track such a temporal change in the distribution of visual attention and update the placement of items accordingly. However, in this case, the system will produce different placements according to the order of the two focused items and need more time to reflect the viewer’s intention in the placement for the second item. Exploring multiple items of different categories in our approach is left as our future research.

In the proposed scenario, we assume that the users do not decide which items they really want at first and need some kind of association to find their favorites by looking at related items as a hint. We interviewed several participants after the eye-tracking experiment to find out the validity of such an assumption. They mostly liked such an idea of encouraging users to identify what they really want through a visual exploration of items. Furthermore, some participants suggested that we can potentially find more preferred items other than the original one through the proposed gaze-based interaction. Other participants argued that this type of interaction could lead to a novel type of recommendation system that effectively helps us make reasonable decisions.

Visually tracking dynamic placements can potentially incur unwanted perceptual stress for users. This perceptual issue has been alleviated, as described previously, by leaving a set of focused items untouched (cf. (D1)) and animating the replacement of other items through a cross-dissolve transition. Moreover, participants in the eye-tracking study also emphasized selecting the proper speed in updating the contents on display. This suggests that users will be comfortable in their exploration if they have sufficient time to look at the items before they are replaced with the next set of items. Pursuing a perceptually plausible timing for updating the placement of items will be an interesting theme, although this is beyond the scope of this research.

The proposed approach will inspire us to develop new techniques for arranging visual symbols, such as icons and pictograms. In particular, we can incorporate the underlying spatial layout of such symbols as constraints into our approach to maximally respect their expected placement. An interesting example is aligning cartographic symbols at a grid while respecting their original geographic positions. This type of visualization method is beneficial because the grid layout of items often leads to aesthetically pleasing representations and improves the readability of the associated visual information (Wood et al. 2011; Cano et al. 2015). In particular, our approach facilitates visualizing spatiotemporal trends inherent in the geospatial data while respecting the underlying temporal context.

Scalability in terms of the number of items is another critical issue. Since we have newly introduced matrix factorization in the topic-based text analysis, we can compose the context map even when we have many more items. This consideration leads us to the idea of first classifying the items into several categories, then selecting the representative items from each group, and finally arranging them in the placement. This hierarchical mechanism provides the viewers with a gaze-driven interface for exploring across multiple levels of detail on demand. We can also strive to pursue the maximum use of our interface to collect different types of data that help us satisfy users’ specific preferences. More extensive integration of our approach with state-of-the-art machine learning techniques is also an exciting theme for future research.

## Conclusion

We have presented an approach for optimizing the dynamic placement of items by respecting the ongoing search context of the viewers. Our gaze-driven system first obtained the spatiotemporal distribution of eye gaze points over the display using an eye-tracker device. It then maximally reflected the personal preferences of the viewers by referring to association rules among the items for more proactive visual exploration. We introduced a context map that maintained the association rules as the mutual relationship between items on a 2D diagram. For this purpose, we applied NMF-based topic modeling techniques to annotated texts associated with the items and introduced dimensionality reduction to the corresponding set of high-dimensional feature vectors. We also demonstrated experimental results and justified the choice of our design criteria through user studies, followed by a discussion on the limitations and future extensions of this work.
